# The effect of steamed potato-wheat bread intake on weight, lipids, glucose, and urinary Na^+^/K^+^: A randomized controlled trial in Chinese adults

**DOI:** 10.3389/fnut.2022.987285

**Published:** 2022-08-25

**Authors:** Haiquan Xu, Yanzhi Guo, Shaolun Cai, Xiuli Wang, Junling Qu, Yunqian Ma, Hongyun Fang, Junmao Sun

**Affiliations:** ^1^Institute of Food and Nutrition Development, Ministry of Agriculture and Rural Affairs, Beijing, China; ^2^National Institute for Nutrition and Health, Chinese Center for Disease Control and Prevention, Beijing, China

**Keywords:** potato staple food, potato bread, glucose, lipids, BMI, urinary Na^+^/K^+^

## Abstract

Steamed potato bread has received much attention from nutritionists and agriculturalists since it became a staple food of China in 2015. Epidemiological studies have indicated that potatoes may cause diabetes and hypertension, but few trials have evaluated this effect. Through a clinical trial, we evaluated the effect of steamed potato bread intake on adults. In total, 49 and 30 individuals were assigned to the intervention and control groups, respectively. Potato-wheat bread (raw wheat flour and cooked potato flour in the ratio 3:7) and steamed wheat bread (100% raw wheat flour) were provided to the intervention and control groups, respectively, once a day for 4 weeks. Compared with the control group, the intervention group showed significant net changes in weight (−0.6 kg; 95% confidence interval [CI]: −1.2, −0.1; *p* = 0.016), body mass index (BMI, −0.2 kg/m^2^; 95% CI: −0.4, −0.1; *p* = 0.020), low-density lipoprotein cholesterol (LDL-c, −0.22 mmol/L; 95% CI: −0.49, −0.01; *p* = 0.035), and the urinary level of Na^+^/K^+^ (−2.4; 95% CI: −4.1, −0.7; *p* = 0.007). In conclusion, the steamed potato-wheat bread intake for 4 weeks resulted in decreases in weight, BMI, LDL-c, and the urinary Na^+^/K^+^ level among Chinese adults.

## Introduction

Potato is one of the most essential food crops, and more than 1 billion people consume it worldwide (1). Although potato consumption is currently the highest in Western countries, it is rapidly becoming a staple food in some other regions. For example, in China, potato has received much attention from nutritionists and agriculturalists recently. In 2015, potato was promoted as a staple food through a food policy and has been included in the Dietary Guidelines for Chinese Residents (2016 edition) (2).

Although potatoes are considered healthy and nutritious, they have a high glycemic index (GI) and glucose load (GL) (3, 4). Some studies have noted a significant association of high GI diet and GL with an increased risk of type 2 diabetes (T2D) (5–7). Furthermore, a meta-analysis revealed a significantly positive association between high consumption of potatoes and risk of T2D, especially through the consumption of French fries (8). Moreover, starch in potatoes becomes digestible on heating, which can raise glucose levels (9).

Since 2015, various food processing enterprises and scientific research institutes have actively invested in using potatoes in noodles, rice noodles, bread, biscuits, and other foods that are part of the Chinese diet. The production of staple foods with potatoes has increased to make foods nutritious. Since the potato staple food policy being proposed by China, many researches have demonstrated the feasibility of this policy (10, 11). However, the effect of potato as a staple food on Chinese people is lacking. Among adolescents, consumption of potato staple food positively affected the total cholesterol (CHO) and insulin profiles but negatively affected the systolic blood pressure (SBP) and high-density lipoprotein cholesterol (HDL-c) (12). However, no trial has been conducted to evaluate the effect of Chinese potato staple food intake among adults, especially in the form of secondary processing production. Hence, more studies are required to determine the effect of potato staple food on human health. Glucose and lipids were hypothesized to not increase abnormally and the urinary Na^+^/K^+^ levels were hypothesized to decrease after frequent intake of potato-wheat bread in adults. Then, this study assessed the effect of steamed potato-wheat bread intake on blood glucose, lipids, blood pressure, and urinary Na^+^/K^+^ in adults. So, this study may provide theoretical support for the policy from health effect perspective.

## Materials and methods

### Study design

**Figure 1:**
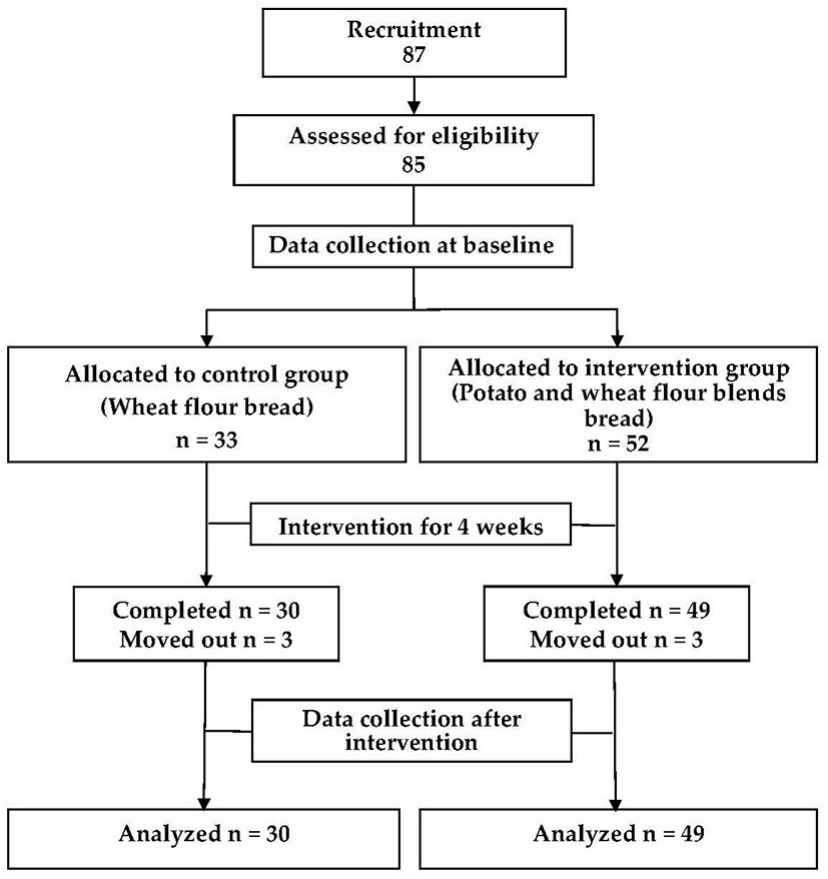
CONSORT flowchart of the study.

An intervention trial was designed. Adult participants were recruited by using posters and social media (from September 26 to October 23, 2016) from Chinese Academy of Agricultural Science. The participants were allocated to either the intervention group (52 subjects) or control group (33 subjects) randomly in accordance with a random number table, 3 participants in intervention and 3 participants in control moved out during the study. In total, 79 participants were analyzed in the study (30 in the control group and 49 in the intervention group). The intervention group was provided steamed potato-wheat bread produced through blending raw wheat flour and cooked potato flour, and their counterparts in the control group were provided steamed bread produced by using raw wheat flour only. Dehydrated cooked potato flour was blended with wheat flour at 30% by weight to make steamed potato-wheat bread (raw wheat flour and cooked potato flour in the ratio 3:7). The baseline data was collected in 28 October 2016, and the final physical examination was performed after 4 weeks' intervention. During the intervention, steamed bread was consumed as staple food at lunch once every day. Other foods were provided to both groups as usual by the research group, both intervention and control participants could choose the vegetables from the supplied category except bread. Figure 1 illustrates the flow of the trial. This trial was registered with the Chinese Clinical Trial Registry (ChiCTR1900027027).

Participants were excluded from the study if (I) they had serious illnesses (e.g., congenital heart disease and kidney disease), (II) they had potato or wheat flour allergies, (III) they could not withstand daily steamed bread intake for 4 weeks, or (IV) they had recently participated in similar interventions.

This trial was conducted in accordance with the Declaration of Helsinki and was approved by the China Ethics Committee of Registering Clinical Trials (Approval Number: ChiECRCT20190210). The informed consent document was voluntarily signed by participants.

### Assessment of intervention effects

Some anthropometric measurements were obtained and urine and blood indictors were evaluated both at baseline and at the end of the intervention. Fasting body weight was measured to the nearest 0.1 kg using a digital scale (RGT-140, Wujin Hengqi Co. Ltd., Changzhou, China), and height was measured using a stadiometer HP-M (Tsutsumi, Tokyo, Japan). The participants did not wear shoes or overcoats during these measurements. Body mass index (BMI) was calculated as weight in kilograms divided by height in meters squared (kg/m^2^). Blood pressure was measured by trained nurses to the nearest 2 mmHg using a mercury sphygmomanometer; during this measurement, patients were in sitting position with at least 10 min of rest before the measurement. SBP and diastolic blood pressure (DBP) were based on the first and fifth Korotkoff sounds, respectively. Two measurements were collected for all participants at 10 min intervals, and the average values were used for the analysis.

Fasting venous blood samples (5 mL) and urine samples (10 mL) were collected in the morning after 10–14 h of overnight fasting. Serum glucose (GLU) was determined through the glucose-oxidase method (Daiichi Pharmaceutical Co., Ltd, Tokyo, Japan) within 4 h after the sample was obtained. CHO, triglycerides (TG), low-density lipoprotein cholesterol (LDL-c), and HDL-c were determined through enzymatic methods by using commercial kits (Daiichi Pharmaceutical Co., Ltd, Tokyo, Japan). Serum insulin was determined through the AxSYM assay based on microparticle enzyme immunoassay technology. Urine samples were collected at the first voiding after waking in the morning for measuring urinary sodium and potassium concentrations. Urinary Na^+^ and K^+^ concentrations (mmol/L) were measured using an ion-specific electrode method, and then the Na^+^/K^+^ molar ratio was calculated.

Analytical methods for different nutrients in bread included direct drying (water), the Kjeldahl method (protein), the Soxhlet extractor method (fat), enzymatic gravimetry (fiber), fluorometry (vitamin C), high-performance liquid chromatography (β-carotene, vitamin E, vitamin B_1_, and vitamin B_2_), the ignition weight method (ash), inductively coupled plasma optical emission spectrometry (sodium, potassium, calcium, iron, and zinc), and the calculation method (energy and carbohydrate). The nutritional analysis indicated that steamed potato-wheat bread provided less energy, protein, fat, carbohydrate, sodium, and iron but more fiber, vitamin E, vitamin B_1_, vitamin B_2_, ash, potassium, calcium, and zinc than the wheat bread (Supplementary Table 1). The bread intake was measured using a daily food record. The diet and activities of the participants during the intervention period were collected with questionnaire including “whether there is any change in your diet?”, “whether you have taken any measures to control your weight?” and “activities and exercises in your leisure time.”

### Statistical analysis

According to the power calculation, a minimum of 26 participants was required for each group for 90% power to detect the effect with a two-sided significance level of 0.05, which was based on the lipid parameter from an intervention study among adolescents (12).

The average daily bread intake was calculated as the indicator of bread intake for participants; then, the energy and nutrients provided by steamed bread were analyzed based on the average bread intake and nutritional content of the bread. The continuous variables (such as age, height, weight BMI and so on) were expressed as mean and standard deviation, and binary variables (such as proportion of sex, proportion of nation) were expressed as sample and percentage. The *t* and chi-square tests were used to compare differences in baseline characteristics between control and intervention groups. The paired *t*-test was used for within-group comparison. The linear mixed effects model was used to compare the changes in continuous variables from the baseline to the end of the study between the control and intervention groups overall after adjusting for confounding factors, including sex, energy intake and physical activity. The intervention effects values were estimated according to Beta values, and 95% confidence interval was then calculated. The effects in subgroups of males and females were analyzed with same model and method. The statistical significance level was set at *p* < 0.05. The SAS software package version 9.2 (SAS Institute Inc, Cary, NC, USA) was used for analysis.

## Results

### General characteristics

The average age of participants was 41.0 ± 8.6 years for the control group and 42.8 ± 8.2 years for the intervention group. The proportions of female participants were 53.3 and 71.4% in the control and intervention groups, respectively. Although the weight of the control group at baseline was more than that of the intervention group, no significant difference in BMI was observed between the two groups. The intervention group consumed significantly more bread than their counterparts did, but the energy provided by the steamed bread daily was not significantly different (Table 1). Compared with the control group, the intervention group received significantly more fiber, vitamin E, vitamin B_1_, vitamin B_2_, potassium, and calcium and less sodium than the control group (Table 2). There's no significant difference between intervention and control groups for the proportion of subjects without changes in diet (60.9% vs. 80.0%, *p* = 0.181), proportion of subjects with weight uncontrolled (80.4% vs. 93.3%, *p* = 0.389), and the average activity time (0.3 ± 0.3 vs. 0.4 ± 0.3, hours / day, *p* = 0.411) during the intervention period.

**Table 1:** Characteristics of participants at baseline.

**Characteristics**	**Control group**	**Intervention group**
Total (*N*)	30	49
**Sex [*****N*** **(%)]**^#^
Male	14 (46.7)	14 (28.6)
Female	16 (53.3)	35 (71.4)
**Nation [*****N*** **(%)]**^#^
Han people	26 (86.7)	46 (93.9)
Minority	4 (13.3)	3 (6.1)
Age [year, Mean (SD)]^†^	41.0 (8.6)	42.8 (8.2)
Height [cm, Mean (SD)]^†^	165.7 (8.4)	163.1 (6.5)
Weight [kg, Mean (SD)]^†^	64.7 (12.5)	62.9 (11.4) ^*^
BMI [kg/m^2^, Mean (SD)]^†^	23.4 (3.0)	23.5 (3.3)
Bread intake [g/day	113.8 (50.3)	136.9 (34.9) ^*^
Mean (SD)]^†^

* *p <0.05.*

# *Comparison based on chi-square test.*

† *Comparison based on t-test. BMI, body mass index*.

**Table 2:** Nutrients provided by the daily steamed breads (mean ± SD).

**Nutrients**	**Control group**	**Intervention group**	***P-*Value**
Energy (kcal)	278.4 ± 123.1	301.2 ± 76.9	0.313
Protein (g)	9.5 ± 4.2	8.7 ± 2.2	0.355
Fat (g)	1.9 ± 0.8	1.7 ± 0.4	0.217
Carbohydrate (g)	54.6 ± 24.1	61.1 ± 15.6	0.194
Fiber (g)	0.6 ± 0.3	1.2 ± 0.3	<0.001
Vitamin E (mg)	0.80 ± 0.35	1.24 ± 0.32	<0.001
Vitamin B1 (mg)	0.08 ± 0.04	0.12 ± 0.03	<0.001
Vitamin B2 (mg)	0.02 ± 0.01	0.07 ± 0.02	<0.001
Sodium (mg)	223.76 ± 98.96	14.04 ± 3.58	<0.001
Potassium (mg)	148.94 ± 65.87	429.03 ± 109.47	<0.001
Calcium (mg)	16.81 ± 7.44	19.76 ± 5.04	0.062
Iron (mg)	1.13 ± 0.50	1.19 ± 0.30	0.582
Zinc (mg)	0.47 ± 0.21	0.64 ± 0.16	0.133

*The t-test was used for the comparison between groups*.

### Physical measurement

**Figure 2:**
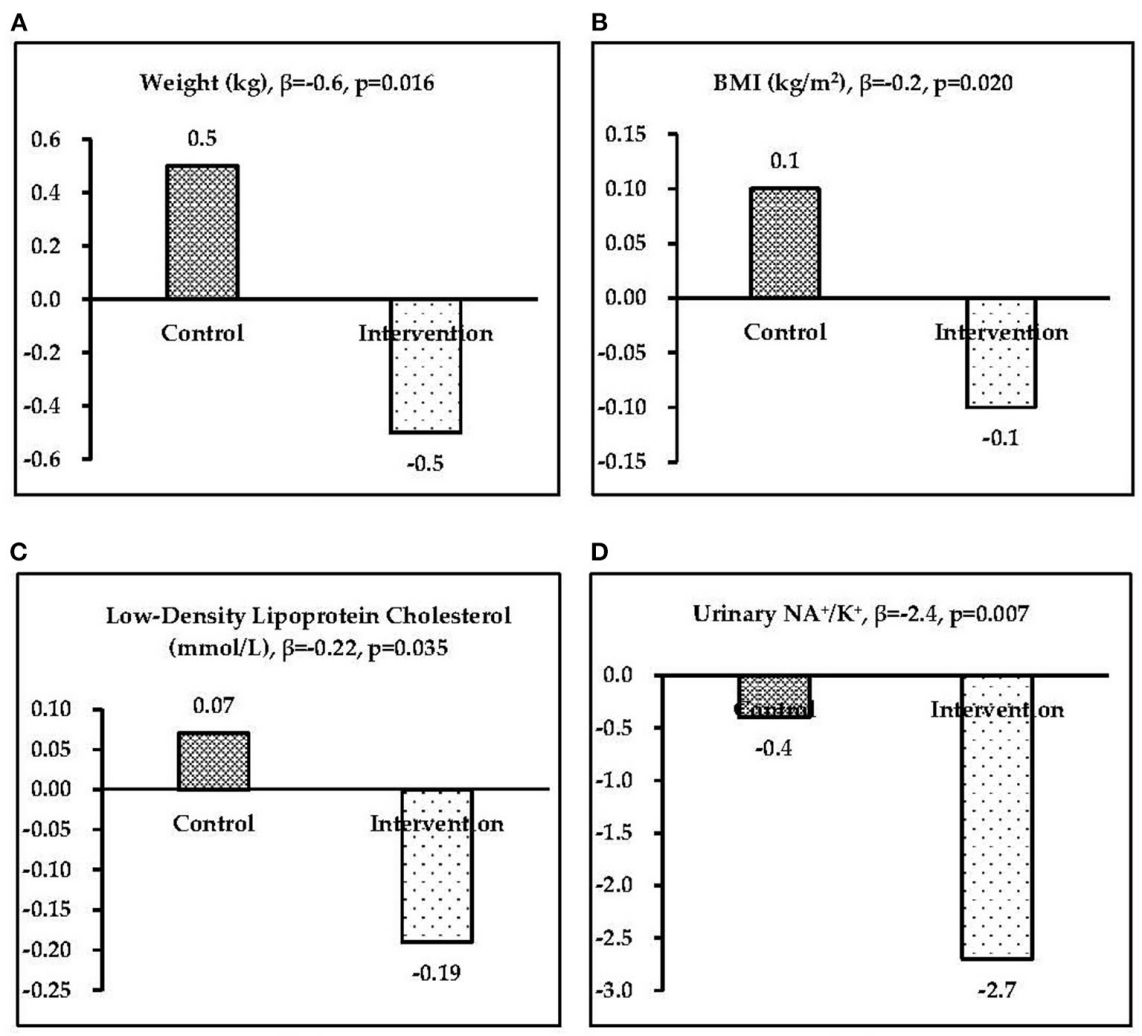
Significant intervention effects (β) in the two groups: **(A)** weight, **(B)** body mass index, **(C)** low-density lipoprotein cholesterol, and **(D)** urinary Na^+^/K^+^.

At baseline, the weight and BMI were 64.7 kg and 23.4 kg/m^2^, respectively, for the control group and 62.9 kg and 23.5 kg/m^2^, respectively, for the intervention group. Compared with the control group, the net changes in weight and BMI for the intervention group after 4 weeks of bread intake were −0.6 kg (95% confidence interval [CI]: −1.2, −0.1; *p* = 0.016) and −0.2 kg/m^2^ (95% CI: −0.4, −0.1; *p* = 0.020), respectively. The SBP and DBP were 121.6 and 75.1 mmHg, respectively, for the control group and 121.2 and 72.3 mmHg, respectively, for the intervention group. Net blood pressure changes in the intervention group were as follows: SBP increased by 0.1 mmHg (95% CI: −4.9, 5.1; *p* = 0.967) and DBP increased by 2.8 mmHg (95% CI: −0.6, 6.2; *p* = 0.100). For the sex-based subgroup analysis, net changes in SBP were 2.2 mmHg (95% CI: −4.9, 9.3; *p* = 0.532) and −0.7 mmHg (95% CI: −7.9, 6.5; *p* = 0.851) for male and female participants, respectively, and the net changes in DBP were 5.3 mmHg (95% CI: 0.4, 10.1; *p* = 0.034) and 1.2 mmHg (95% CI: −3.6, 6.0; *p* = 0.624) among male and female participants, respectively (Table 3, Figure 2).

**Table 3:** Outcomes of the intervention for groups and subgroups.

**Subgroups**	**Variable**	**Control group**		**Intervention group**		**Effect**	
		**Baseline**	**End**	**Baseline**	**End**	**Beta (95% CI)**	***P*-Value**
Overall	Weight (kg)	64.7 ± 12.5	65.2 ± 12.7**	62.9 ± 11.4	62.6 ± 11	−0.6 (−1.2, −0.1)	0.016
	BMI (kg/m^2^)	23.4 ± 3.1	23.5 ± 3.1**	23.5 ± 3.3	23.4 ± 3.1	−0.2 (−0.4, −0.1)	0.020
	SBP (mmHg)	121.6 ± 13.8	120.2 ± 14.9	121.2 ± 15.9	120 ± 16.3	0.1 (−4.9, 5.1)	0.967
	DBP (mmHg)	75.1 ± 11.1	70.3 ± 9.3**	72.3 ± 11.2	70.2 ± 10.8	2.8 (−0.6, 6.2)	0.100
	GLU (mmol/L)	5.29 ± 0.58	5.37 ± 0.55	5.10 ± 0.47	5.12 ± 0.50	−0.01 (−0.39, 0.07)	0.900
	INS (mIU/L)	11.19 ± 3.17	13.17 ± 3.81**	9.91 ± 6.18	12.94 ± 6.12**	1.22 (−0.49, 2.93)	0.158
	CHO (mmol/L)	4.8 ± 0.82	4.86 ± 0.89	4.71 ± 0.78	4.58 ± 0.80	−0.17 (−0.43, 0.09)	0.194
	TG (mmol/L)	1.29 ± 1.02	1.62 ± 2.33	1.07 ± 0.47	1.16 ± 0.54	−0.22 (−0.80, 0.35)	0.440
	LDL-c (mmol/L)	3.08 ± 0.87	3.15 ± 0.87	2.86 ± 0.8	2.67 ± 0.78**	−0.22 (−0.49, −0.01)	0.035
	HDL-c (mmol/L)	1.44 ± 0.28	1.47 ± 0.34	1.61 ± 0.36	1.58 ± 0.37	−0.24 (−0.77, 0.29)	0.371
	Urinary Na^+^ (mmol/L)	129.4 ± 34.5	141.5 ± 43.4	134.3 ± 39.5	138.8 ± 35.7	−7.1 (−29.1, 14.9)	0.520
	Urinary K^+^ (mmol/L)	39.7 ± 27.0	48.0 ± 33.5	31.5 ± 20.4	54.1 ± 35.5**	14.4 (−4.7, 33.5)	0.136
	Urinary Na^+^/K^+^	4.7 ± 2.8	4.3 ± 2.5	6.0 ± 3.6	3.3 ± 1.7**	−2.4 (−4.1, −0.7)	0.007
Male	Weight (kg)	74.5 ± 8.6	75.1 ± 8.7*	73.3 ± 11.8	73.0 ± 11.1	−0.7 (−1.9, 0.4)	0.178
	BMI (kg/m^2^)	25 ± 2.4	25.2 ± 2.4*	25.6 ± 3.5	25.5 ± 3.3	−0.3 (−0.6, 0.1)	0.093
	SBP (mmHg)	126.1 ± 12.5	124.8 ± 13.4	124.3 ± 17.1	125.2 ± 12.2	2.2 (−4.9, 9.3)	0.532
	DBP (mmHg)	79.9 ± 10.5	73.7 ± 9.5**	74.5 ± 11.9	73.5 ± 10.2	5.3 (0.4, 10.1)	0.034
	GLU (mmol/L)	5.38 ± 0.38	5.44 ± 0.4	5.49 ± 0.39	5.5 ± 0.54	0.06 (−0.28, 0.40)	0.727
	INS (mIU/L)	11.57 ± 2.52	13.85 ± 3**	12.5 ± 8.71	16.49 ± 8.94*	1.72 (−2.29, 5.73)	0.386
	CHO (mmol/L)	4.88 ± 0.74	4.84 ± 0.78	4.82 ± 0.92	4.7 ± 0.85	0.01 (−0.42, 0.44)	0.955
	TG (mmol/L)	1.46 ± 1.14	1.38 ± 0.74	1.27 ± 0.61	1.34 ± 0.71	0.18 (−0.42, 0.77)	0.545
	LDL-c (mmol/L)	3.19 ± 0.78	3.22 ± 0.89	3.07 ± 0.97	2.87 ± 0.84	−0.14 (−0.65, 0.36)	0.567
	HDL-c (mmol/L)	1.39 ± 0.34	1.38 ± 0.32	1.44 ± 0.3	1.45 ± 0.25	0.02 (−0.13, 0.17)	0.778
	Urinary Na^+^ (mmol/L)	135.3 ± 31.1	136.3 ± 44.7	140.8 ± 44.5	140.6 ± 44.5	−1.2 (−43.6, 41.2)	0.954
	Urinary K^+^ (mmol/L)	48.5 ± 30.3	44.9 ± 37.1	31.8 ± 19.5	42.6 ± 19.6	14.4 (−17.6, 46.5)	0.364
	Urinary Na^+^/K^+^	3.9 ± 2.4	4.6 ± 2.6	5.9 ± 3.1	3.8 ± 1.8**	−2.9 (−5.6, −0.1)	0.039
Female	Weight (kg)	56.1 ± 8.3	56.5 ± 8.5	58.7 ± 8.2	58.4 ± 7.8	−0.5 (−1.1, 0)	0.072
	BMI (kg/m^2^)	22 ± 2.9	22.1 ± 2.9	22.7 ± 2.9	22.6 ± 2.7	−0.3 (−0.6, 0)	0.048
	SBP (mmHg)	117.6 ± 14.1	116.2 ± 15.3	120 ± 15.5	117.9 ± 17.4	−0.7 (−7.9, 6.5)	0.851
	DBP (mmHg)	70.9 ± 10.1	67.3 ± 8.4*	71.4 ± 11	68.9 ± 11	1.2 (−3.6, 6.0)	0.624
	GLU (mmol/L)	5.22 ± 0.71	5.3 ± 0.66	4.94 ± 0.41	4.97 ± 0.39	−0.05 (−0.28, 0.19)	0.689
	INS (mIU/L)	10.86 ± 3.7	12.57 ± 4.41**	8.88 ± 4.58	11.52 ± 3.87**	0.99 (−0.44, 2.41)	0.171
	CHO (mmol/L)	4.74 ± 0.91	4.89 ± 0.99	4.66 ± 0.72	4.53 ± 0.78	−0.29 (−0.63, 0.05)	0.097
	TG (mmol/L)	1.15 ± 0.92	1.83 ± 3.15	0.99 ± 0.38	1.08 ± 0.44	−0.58 (−1.44, 0.29)	0.184
	LDL-c (mmol/L)	2.98 ± 0.96	3.09 ± 0.88	2.78 ± 0.72	2.59 ± 0.75*	−0.28 (−0.55, −0.01)	0.049
	HDL-c (mmol/L)	1.49 ± 0.21	1.54 ± 0.34	1.68 ± 0.36	1.64 ± 0.4	−0.10 (−0.25, 0.05)	0.176
	Urinary Na^+^ (mmol/L)	123.9 ± 37.6	146.4 ± 43.1	131.5 ± 37.6	137.9 ± 31.7	−14.7 (−41, 11.6)	0.265
	Urinary K^+^ (mmol/L)	31.5 ± 21.3	50.9 ± 30.8*	31.5 ± 21.1	59.4 ± 40.0**	8.9 (−15.7, 33.5)	0.470
	Urinary Na^+^/K^+^	5.4 ± 3.0	4.2 ± 2.5	6.0 ± 3.8	3.1 ± 1.6**	−1.7 (−4.0, 0.7)	0.162

The linear mixed effects model was used for comparing the groups. The comparison of overall participants was adjusted for sex, energy intake and physical activity. The paired t-test was used for within-group comparison; ^**^p <0.01; ^*^p <0.05. BMI, body mass index; CI, confidence interval; SBP, systolic blood pressure; DBP, diastolic blood pressure; GLU, serum glucose; INS, serum insulin; CHO, total cholesterol; HDL-c, high-density lipoprotein cholesterol; LDL-c, low-density lipoprotein cholesterol; TG, triglycerides.

### Blood indicators

Compared with the control group, both lipid metabolic indicators (i.e., CHO, TG, HDL-c, and LDL-c) and blood glucose exhibited a decreasing trend in the intervention group, and a significant net change (mean between-group difference) of −0.22 mmol/L for LDL-c (95% CI: −0.49, −0.01; *p* = 0.035) was observed in the intervention group after 4 weeks of potato bread intake (Figure 2). After the sex-based subgroup analysis, a significant net change in LDL-c of −0.28 mmol/L (95% CI: −0.55, −0.01; *p* = 0.049) was observed in female participants, but male participants exhibited no significant changes. For GLU, a decreasing trend were observed −0.05 mmol/L (95% CI: −0.28, 0.19; *p* = 0.689) in intervention women (Table 3).

### Urinary sodium and potassium

Compared with the control group, urinary Na^+^ and K^+^ excretions in the intervention group changed by −7.1 (95% CI: −29.1, 14.9; *p* = 0.520) mmol/L and 14.4 (95% CI: −4.7, 33.5; *p* = 0.136) mmol/L, respectively. Significant changes in the Na^+^/K^+^ ratio were observed of −2.4 (95% CI: −4.1, −0.7; *p* = 0.007) overall, −2.9 (95% CI: −5.6, −0.1; *p* = 0.039) among men, and −1.7 (95% CI: −4.0, 0.7; *p* = 0.162) among women (Table 3).

## Discussion

To the best of our knowledge, this is the first trial to evaluate the effect of steamed potato-wheat bread intake on Chinese adults. The results revealed significant effects on weight, BMI, LDL-c, and the urinary Na^+^/K^+^ ratio after the intake of steamed potato-wheat bread as staple food once daily for 4 weeks in this study. Compared with the wheat bread group, the potato-wheat bread group showed a significant net decrease in weight, BMI, LDL-c, and the urinary Na^+^/K^+^ level.

Vitamin C is often lacking in the diet of individuals without access to fresh produce. Although vitamin C is destroyed during the cooking process and potatoes have a moderate content of vitamin C compared with some other fruits and vegetables, potatoes play a critical nutritional role as the primary source of vitamin C in many countries, as well as providing fiber, potassium, calcium, iron, vitamin B_6_, niacin, and folate, which are related to a reduced risk of several chronic diseases (13). The importance of potatoes for vitamin C intake is partly because they can be stored, but the Chinese steamed potato-wheat bread was made from a mixture of cooked potato flour and wheat flour, and most vitamin C was destroyed while processing the bread; thus, little vitamin C was left in the steamed potato bread (<0.044 mg/100 g). Compared with wheat staple food, potato staple bread contains more potassium and less sodium, which is associated with cardiovascular health, may control hypertension, and decreases the urinary Na^+^/K^+^ level.

Adiposity and potato consumption have been positively associated in previous trials (14, 15); more specifically, the intake of boiled, baked, and mashed potatoes was associated with a slight increase in body weight in both sexes (14) and an increase in waist circumference in women (15). However, in this trial, compared with the wheat bread group (control group), the weight and BMI decreased in potato-wheat bread group (intervention group). The positive associations between potatoes and body weight from cohort studies may have been confounded by unmeasured factors of unhealthy lifestyle (16). Furthermore, French fries showed a stronger association with weight gain than intake of other potato foods (14, 17). This may be due to the higher energy density of French fries compared with baked, boiled, and mashed potatoes (18) and the unhealthy lifestyle that may be related to the intake of French fries (19). Another study reported that consumption of French fries is associated with a higher mortality risk, but total potato consumption was not associated with mortality (20). For the Chinese staple potato bread was processed by using the steaming method, and the potato bread provided less energy than the wheat bread (230.8 kcal/100 g vs. 248.5 kcal/100 g). Although the intervention group received more energy than the control group, the weight of the intervention group appeared to relatively decrease.

Beyond the nutritional properties of a quality staple food, potato may have a role to play in human health over a lifetime of consumption. Potato has been implicated in contributing to diabetes because of its high GI and GL (5, 21). Studies on potatoes have shown GI values as high as 118 (22) and have associated high GI and GL with an increased risk of several chronic diseases (23–25). Moreover, diets rich in potatoes are commonly unhealthy (20). The nutrient content of potatoes is dependent on the cooking method (26, 27). Fried potatoes typically contain high amounts of fats and salts. Boiling, baking, and microwaving can reduce vitamin C, thiamin, riboflavin, niacin, folic acid, and vitamin B_6_ in potatoes. Roasting or baking could enhance the availability of minerals in potatoes. Heating potatoes converts their native starch granules to rapidly digestible starch (28). Hence, boiled potatoes have higher GI than potatoes cooked using other methods. Therefore, the effects of potato consumption are greatly dependent on the type of potato product consumed.

Some epidemiological studies have shown that potatoes are positively associated with T2D (6, 7) and gestational diabetes mellitus (29). In a cross-sectional study involving 4,774 Iranian adults, the frequency of potato consumption was associated with high fasting blood glucose level (30) and T2D. Moreover, three prospective studies in the USA have reported that high potato consumption was associated with a high risk of diabetes mellitus and hypertension (31, 32). Thus, potato consumption has a significant role in the progression of T2D. Unlike other vegetables, potatoes have a high GI and are rich in starch, which is absorbed rapidly (22, 33). High potato consumption may lead to a sharp increase in postprandial blood glucose concentrations, resulting in β-cell dysfunction or exhaustion and T2D (34–36). However, some prospective studies have reported that potato consumption is not associated with the risk of incident cardiovascular disease (37) and hypertension (38). One prospective cohort consisting of 64,227 Chinese women without a history of T2D found that the risk of T2D was reduced by 28% in the category with the highest consumption of potatoes versus the category with the lowest potato consumption (39). Increased potato consumption during a 20-year follow-up study was inversely related to 2-h glucose level (40). Furthermore, the increasing effect on glucose level was not observed in this trial, possibly because the processed method was used for the potato staple bread. The potato flour used to produce the steamed potato bread was made from cooked potato. Previous researches indicated that repeated cooking can increase the resistant starch in potato by 10% (41, 42). After a secondary heating treatment with a cooling interval, the proportion of resistant starch in steamed potato bread might increase. Resistant starch reportedly plays a role in controlling blood glucose and insulin levels (43, 44). Additionally, the different potato cultivars from different areas may play a role.

The Chinese potato stapling strategy is closely related to agriculture, nutrition, and public health. A novel potato cultivar was needed for the staple bread. A novel potato cultivar for potato staple food should be bred and selected based on certain traits, such as high dry weight, which is one of the most essential traits for staple food processing. However, based on the possible relationship between potato consumption and T2D, a high resistance starch cultivar maybe one target. Biotechnology-based tools are now widely used to enhance and expand the traditional remit of potatoes in food production. Enabling potatoes to produce therapeutic compounds through modification of their functionality is now a reality. In this review, strong corporation from nutritionists, agriculturalists, and public health researchers will be needed to improve this crop. Moreover, potatoes could be genetically modified to improve the functional properties of tuber-derived flour through the expression of beneficial traits related to the gene (45).

This trial had also some limitations. Firstly, the duration of steamed potato bread intake was short; Secondly, there was a lack of dietary analysis and digestibility measures; Thirdly, though main confounding factors have been controlled, still some factors can't be controlled about the population, and subgroups were not considered in the sample design; Lastly, the concentration in a spot voiding urine not 24-h urinary excretion for sodium and potassium may cause the results inaccurate. All these factors may have affected the results. However, this is the first clinical trial to evaluate the effect of steamed potato bread on blood pressure, glucose, lipids, and urinary Na^+^ and K^+^ in healthy Chinese adults, and it could provide valuable evaluation data for the potato stapling strategy in China.

## Conclusion

The intake of steamed potato bread, which was made from a blend of wheat flour and cooked potato flour, once daily for 4 weeks in adults could decrease weight, BMI, LDL-c, and the urinary Na^+^/K^+^ level, and especially increase DBP in male participants.

## Data availability statement

The original contributions presented in the study are included in the article/Supplementary material, further inquiries can be directed to the corresponding author/s.

## Author contributions

HX led the development of the manuscript and primary data analysis. HX, YG, HF, and JS contributed to the research design and trial methodology. HX, XW, YM, JQ, and SC contributed to the sample data collection. All authors contributed to the revision and approval of the final content of the manuscript.

## Funding

This study was supported by the Special Research Grant for Nonprofit Public Service of China (Agriculture) (grant number 201503001) and Science and Technology Innovation Project of the Chinese Academy of Agricultural Science (grant number ASTIP2022).

## Conflict of interest

The authors declare that the research was conducted in the absence of any commercial or financial relationships that could be construed as a potential conflict of interest.

## Publisher's note

All claims expressed in this article are solely those of the authors and do not necessarily represent those of their affiliated organizations, or those of the publisher, the editors and the reviewers. Any product that may be evaluated in this article, or claim that may be made by its manufacturer, is not guaranteed or endorsed by the publisher.

## References

[1] Hermansen A Lu D Forbes G. Potato production in China and Norway: similarities, differences and future challenges. Potato Res. (2012) 55:197–203. 10.1007/s11540-012-9224-7

[2] Chinese Nutrition Society. Dietary Guidelines for Chinese. Beijing: People's Medical Publication House (2017). p. 362.

[3] Nayak B Berrios JJ Tang J. Impact of food processing on the glycemic index (GI) of potato products. Food Res Int. (2014) 56:35–46. 10.1016/j.foodres.2013.12.02010889806

[4] Kusnadi D Barclay AW Brand-Miller JC Louie J. Changes in dietary glycemic index and glycemic load in Australian adults from 1995 to 2012. Am J Clin Nutr. (2017) 106:189–98. 10.3945/ajcn.116.150516 28566308

[5] van Bakel MM Kaaks R Feskens EJ Rohrmann S Welch AA Pala V. Dietary glycaemic index and glycaemic load in the European prospective investigation into cancer and nutrition. Eur J Clin Nutr. (2009) 63:188–205. 10.1038/ejcn.2009.81 19888274

[6] Salmerón J Manson JE Stampfer MJ Colditz GA Wing AL Willett WC. Dietary fiber, glycemic load, and risk of non-insulin-dependent diabetes mellitus in women. JAMA. (1997) 277:472–7. 10.1001/jama.277.6.472 9020271

[7] Salmerón J Ascherio A Rimm EB Colditz GA Spiegelman D Jenkins DJ. Dietary fiber, glycemic load, and risk of NIDDM in men. Diabetes Care. (1997) 20:545–50. 10.2337/diacare.20.4.545 9096978

[8] Zhang Y You D Lu N Duan D Feng X Astell-Burt T. Potatoes consumption and risk of type 2 diabetes: a meta-analysis. Iran J Public Health. (2018) 47:1627–35. 30581777 PMC6294859

[9] Augustin L. Glycaemic index in chronic disease. Nutrafoods. (2013) 12:117–25. 10.1007/s13749-013-0061-3

[10] Gao B Huang W Xue X Hu Y Huang Y Wang L. Comprehensive environmental assessment of potato as staple food policy in China. Int J Environ Res Public Health. (2019) 16:2700. 10.3390/ijerph16152700 31362347 PMC6695635

[11] Xu H Wang X Ma G. Nutrition feasibility analysis of development of potato as a staple food (in Chinese). Food Nutr China. (2015) 21:13–17.

[12] Xu H Guo Y Lu S Ma Y Wang X Zhao L. Effect of steamed potato bread intake on glucose, lipids, and urinary Na+ and K+: a randomized controlled trial with adolescents. Int J Environ Res Public Health. (2020) 17:2096. 10.3390/ijerph17062096 32235690 PMC7143724

[13] Darooghegi Mofrad M Djafarian K Mozaffari H Shab-Bidar S. Effect of magnesium supplementation on endothelial function: a systematic review and meta-analysis of randomized controlled trials. Atherosclerosis. (2018) 273:98–105. 10.1016/j.atherosclerosis.2018.04.020 29709832

[14] Mozaffarian D Hao T Rimm E Willett W Hu F. Changes in diet and lifestyle and long-term weight gain in women and men. N Engl J Med. (2011) 364:2392–404. 10.1056/NEJMoa1014296 21696306 PMC3151731

[15] Halkjær J Tjønneland A Overvad K Sørensen TI. Dietary predictors of 5-year changes in waist circumference. J Am Diet Assoc. (2009) 109:1356–66. 10.1016/j.jada.2009.05.015 19631041

[16] Nicklas TA O'Neil C Fulgoni VL. Differing statistical approaches affect the relation between egg consumption, adiposity, and cardiovascular risk factors in adults. J Nutr. (2015) 145:170–6S. 10.3945/jn.114.194068 25527676

[17] Linde JA Utter J Jeffery RW Sherwood NE Pronk NP Boyle RG. Specific food intake, fat and fiber intake, and behavioral correlates of BMI among overweight and obese members of a managed care organization. Int J Behav Nutr Phys Act. (2006) 3:1–8. 10.1186/1479-5868-3-42 17125525 PMC1684256

[18] Camire ME Kubow S Donnelly DJ. Potatoes and human health. Crit Rev Food Sci Nutr. (2009) 49:823–40. 10.1080/10408390903041996 19960391

[19] Langsetmo L Poliquin S Hanley DA Prior JC Barr S Anastassiades T. Dietary patterns in Canadian men and women ages 25 and older: relationship to demographics, body mass index, and bone mineral density. BMC Musculoskelet Disord. (2010) 11:20. 10.1186/1471-2474-11-20 20109205 PMC2835657

[20] Veronese N Stubbs B Noale M Solmi M Vaona A Demurtas J. Fried potato consumption is associated with elevated mortality: an 8-y longitudinal cohort study. Am J Clin Nutr. (2017) 106:162–7. 10.3945/ajcn.117.154872 28592612 PMC5486204

[21] Wirfält E McTaggart A Pala V Gullberg B Frasca G Panico S. Food sources of carbohydrates in a European cohort of adults. Public Health Nutr. (2002) 5:1197–215. 10.1079/PHN2002399 12639227

[22] Atkinson FS Foster-Powell K Brand-Miller JC. International tables of glycaemic index and glycaemic load values. Diabetes Care. (2008) 31:2281–3. 10.2337/dc08-1239 18835944 PMC2584181

[23] Fan J Song Y Wang Y Hui R Zhang W. Dietary glycemic index, glycemic load, and risk of coronary heart disease, stroke, and stroke mortality: a systematic review with meta-analysis. PLoS ONE. (2012) 7:e52182. 10.1371/journal.pone.0052182 23284926 PMC3527433

[24] Mirrahimi A Chiavaroli L Srichaikul K Augustin LS Sievenpiper JL Kendall CW. The role of glycemic index and glycemic load in cardiovascular disease and its risk factors: a review of the recent literature. Curr Atheroscler Rep. (2014) 16:381. 10.1007/s11883-013-0381-1 24271882

[25] Sieri S Krogh V Agnoli C Ricceri F Palli D Masala G. Dietary glycemic index and glycemic load and risk of colorectal cancer: results from the EPIC-Italy study. Int J Cancer. (2015) 136:2923–31. 10.1002/ijc.29341 25403784

[26] Siri-Tarino PW Sun Q Hu FB Krauss RM. Meta-analysis of prospective cohort studies evaluating the association of saturated fat with cardiovascular disease. Am J Clin Nutr. (2010) 91:535–46. 10.3945/ajcn.2009.27725 20071648 PMC2824152

[27] Augustin J Johnson SR Teitzel C True RH Hogan JM Toma RB. Changes in the nutrient composition of potatoes during home preparation: II. Vitamins. Am J Pota Res. (1978) 55:653–62. 10.1007/BF02852138

[28] García-Alonso A Goñi I. Effect of processing on potato starch: *in vitro* availability and glycaemic index. Die Nahrung. (2000) 44:19–22. 10.1002/(SICI)1521-3803(20000101)44:1&lt;19::AID-FOOD19&gt;3.0.CO;2-E 10702994

[29] Bao W Tobias DK Hu FB Chavarro JE Zhang C. Pre-pregnancy potato consumption and risk of gestational diabetes mellitus: prospective cohort study. BMJ. (2016) 352:h6898. 10.1136/bmj.h6898 26759275 PMC5115165

[30] Khosravi-Boroujeni H Mohammadifard N Sar-rafzadegan N Sajjadi F Maghroun M Khosravi A. Potato consump-tion and cardiovascular disease risk factors among Iranian population. Int J Food Sci Nutr. (2012) 63:913–20. 10.3109/09637486.2012.690024 22639829

[31] Muraki I Rimm EB Willett WC Manson JE Hu FB Sun Q. Potato consumption and risk of type 2 diabetes: results from three prospective cohort studies. Diabetes Care. (2016) 39:376–84. 10.2337/dc15-0547 26681722 PMC4764041

[32] Borgi L Rimm EB Willett WC Forman JP. Potato intake and incidence of hypertension: results from three prospective US cohort studies. BMJ. (2016) 353:i2351. 10.1136/bmj.i2351 27189229 PMC4870381

[33] McGill CR Kurilich AC Davignon J. The role of potatoes and potato components in cardiometabolic health: a review. Ann Med. (2013) 45:467–73. 10.3109/07853890.2013.813633 23855880

[34] Riccardi G Rivellese AA Giacco R. Role of glycemic index and glycemic load in the healthy state, in prediabetes, and in diabetes. Am J Clin Nutr. (2008) 87:269–74S. 10.1093/ajcn/87.1.269S 18175767

[35] Bhupathiraju SN Tobias DK Malik VS Pan A Hruby A Manson JE. Glycemic index, glycemic load, and risk of type 2 diabetes: results from 3 large US cohorts and an updated meta-analysis. Am J Clin Nutr. (2014) 100:218–32. 10.3945/ajcn.113.079533 24787496 PMC4144100

[36] Ceriello A Esposito K Piconi L Ihnat MA Thorpe JE Testa R. Oscillating glucose is more deleterious to endothelial function and oxidative stress than mean glucose in normal and type 2 diabetic patients. Diabetes. (2008) 57:1349–54. 10.2337/db08-0063 18299315

[37] Larsson SC Wolk A. Potato consumption and risk of cardiovascular disease: 2 prospective cohort studies. Am J Clin Nutr. (2016) 104:1245–52. 10.3945/ajcn.116.142422 27680993

[38] Hu EA Martinez-Gonzalez MA Salas-Salvado J Corella D Ros E Fito M. Potato consumption does not increase blood pressure or incident hypertension in 2 cohorts of Spanish adults. J Nutr. (2017) 147:2272–81. 10.3945/jn.117.252254 29046405

[39] Villegas R Liu S Gao YT Yang G Li H Zheng W. Prospective study of dietary carbohydrates, glycemic index, glycemic load, and incidence of type 2 diabetes mellitus in middle-aged Chinese women. Arch Intern Med. (2007) 167:2310–6. 10.1001/archinte.167.21.2310 18039989

[40] Feskens EJ Virtanen SM Rasanen L Tuomilehto J Stengård J Pekkanen J. Dietary factors determining diabetes and impaired glucose tolerance. A 20-year follow-up of the Finnish and Dutch cohorts of the seven countries study. Diabetes Care. (1995) 18:1104–12. 10.2337/diacare.18.8.1104 7587845

[41] Meng T Yan Y Zhao C Ye X. Effect of Chinese cooking on the resistant starch and other main nutrient elements in potatoes (in Chinese). Sci Technol Food Industry. (2012) 33:86–9.

[42] Berry CS. Resistant starch: formation and measurement of starch that survives exhaustive digestion with amylolytic enzymes during the determination of dietary fibre. J Cereal Sci. (1986) 4:301–14. 10.1016/S0733-5210(86)80034-0

[43] Raigond P Ezekiel R Raigond B. Resistant starch in food: a review. J Sci Food Agric. (2015) 95:1968–78. 10.1002/jsfa.6966 25331334

[44] Tian J Chen J Ye X Chen S. Health benefits of the potato affected by domestic cooking: a review. Food Chem. (2016) 202:165–75. 10.1016/j.foodchem.2016.01.120 26920281

[45] Mullins E Milbourne D Petti C Doyle-Prestwich BM Meade C. Potato in the age of biotechnology. Trends Plant Sci. (2006) 11:254–60. 10.1016/j.tplants.2006.03.002 16621672

