# Supplementary material for: The effect of steamed potato-wheat bread intake on weight, lipids, glucose, and urinary Na+/K+: A randomized controlled trial in Chinese adults
Source: Front Nutr. 2022 Aug 25;9:987285. doi: 10.3389/fnut.2022.987285 (PMC9453233; doi:10.3389/fnut.2022.987285)
Supplement: Supplementary file 1 [file Table_1.docx]

**TABLE 1 |** Nutritional content of two types of steamed bread (100 g)

| **Nutrients** | **Wheat Bread** | **Potato Bread** |
| --- | --- | --- |
| Energy (kcal) | 248.5 | 230.8 |
| Water (g) | 40.0 | 43.6 |
| Protein (g) | 8.5 | 6.7 |
| Fat (g) | 1.7 | 1.3 |
| Carbohydrate (g) | 48.7 | 46.8 |
| Fiber (g) | 0.5 | 0.9 |
| Vitamin C (mg) | ＜0.044 | ＜0.044 |
| β-Carotene (μg) | ＜2.00 | ＜2.00 |
| Vitamin E (mg) | 0.716 | 0.949 |
| Vitamin B_1_ (mg) | 0.073 | 0.095 |
| Vitamin B_2_ (mg) | 0.02 | 0.05 |
| Ash (g) | 0.64 | 0.69 |
| Sodium (mg) | 199.74 | 10.76 |
| Potassium (mg) | 132.95 | 328.76 |
| Calcium (mg) | 15.01 | 15.14 |
| Iron (mg) | 1.01 | 0.91 |
| Zinc (mg) | 0.42 | 0.49 |
